# Supplementary material for: Towards developing brain-computer interfaces for people with Multiple Sclerosis
Source: PLoS One. 2025 Mar 18;20(3):e0319811. doi: 10.1371/journal.pone.0319811 (PMC11918325; doi:10.1371/journal.pone.0319811)
Supplement: S1 Survey — (PDF) [file pone.0319811.s001.pdf]

PLS

Plain Language Statement
Faculty of Engineering and Information Technology (FEIT)
Department of Biomedical Engineering

Project: Designing Brain-Computer Interfaces for Individuals with Multiple Sclerosis

Responsible Researcher:
Dr. Sam John Tel: +61 3 8344 9437 Email: sam.john@unimelb.edu.au

Additional Researchers:
Mr. Kirill Kokorin Email: kkokorin@student.unimelb.edu.au
Mr. John Russo Email: russoj1@student.unimelb.edu.au
Mr. Tim Mahoney Email: tbmahoney@student.unimelb.edu.au
Dr. Ashley Reynolds Email: ashleyr@student.unimelb.edu.au

What is this research about?
Brain-computer interfaces (BCIs) use sensors to record brain activity and translate that activity into user-intended commands, which can be used to control a device. This project aims to understand what individuals with multiple sclerosis (MS) would like to see in a BCI so that it is most beneficial to them.

What will I be asked to do?
You will be asked to complete a survey which should take you less than 15 minutes. The survey will ask you to describe your experience with MS and review a range of potential BCI system. You will need to complete the entire survey for your responses to be saved.

What are the possible benefits?
By participating in this research, you will inform future designs of BCIs aimed at assisting people with MS.

What are the possible risks?
If describing your experience with MS makes you feel distressed, you may end the survey at any time. All of your responses will be deleted.

Do I have to take part?

No, participation is completely voluntary. The survey is completely anonymous, and you will not be asked to provide any personally identifiable information. You may exit the survey at any time and all of your responses will be deleted immediately.

Will I hear about the results of this project?

The results of this study will be published in scientific literature. Results may also be presented at international and local conferences. Since the survey is anonymous, the researchers are unable to contact you regarding any publication outcomes. If you wish to stay updated with the project, please follow the project page at the end of this survey.

What will happen to information about me?

The survey will not gather any personally identifiable information about you. However, you will be asked to provide your age range. By taking part in the survey, you confirm that you are over 18 years of age. You will not be able to withdraw your survey responses after you have submitted the survey, but you may choose not to proceed with your submission by ending the survey at any time. Survey results will be stored in a secure folder and only available to researchers listed on the ethics approval. All data will be destroyed 5 years after publication. Since the survey is anonymous, you will not be able to request to view your data after submitting the survey.

Who is funding this project?

This project is not funded externally.

Where can I get further information?

If you would like more information about the project, please contact the researchers:
Dr. Sam John sam.john@unimelb.edu.au
Mr. Kirill Kokorin kkokorin@student.unimelb.edu.au

Who can I contact if I have any concerns about the project?

This project has human research ethics approval from The University of Melbourne 26078. If you have any concerns or complaints about the conduct of this research project, which you do not wish to discuss with the research team, you should contact the Research Integrity Administrator, Office of Research Ethics and Integrity, University of Melbourne, VIC 3010. Tel: +61 8344 1814 or Email: research-integrity@unimelb.edu.au. All complaints will be treated confidentially. In any correspondence please provide the name of the research team and/or the name or ethics ID number of the research project.

Please be aware this survey discusses technology aimed toward assisting people whose MS symptoms are severe. Please do not continue the survey if you find these topics confronting.

By proceeding with this survey, you acknowledge that:

1. **You have read and understood this Plain Language Statement,**
2. **you are over 18 years of age and**
3. **you agree to continue this survey.**

- ○ Acknowledge and continue
- ○ Do not continue

You and Your MS

MS can present differently in different people. In this section, we would like to understand you and your condition

How old are you?

- ○ 18 - 29
- ○ 30 - 49
- ○ 50 - 69
- ○ 70+

What country do you currently live in?

____

What is your highest level of education?

- ○ High School or lower
- ○ Tafe Cert or Diploma

- ○ Undergraduate Degree
- ○ Postgraduate Degree

How long have you been diagnosed with MS?

- ○ Less than 1 year
- ○ 1 - 5 years
- ○ 6 - 10 years
- ○ 11 - 20 years
- ○ 21 - 30 years
- ○ 31 - 40 years
- ○ More than 40 years

Is your MS:

- ○ Relapsing Remitting (RRMS)
- ○ Progressive (SPMS/PPMS)
- ○ Unknown
- ○ ________ Other (specify):

What MS symptoms do you experience? Please select all that apply.

- ☐ Cognitive impairment
- ☐ Depression
- ☐ Fatigue
- ☐ Seizures
- ☐ Vertigo
- ☐ Pain
- ☐ Motor symptoms in arms (weakness, paralysis, coordination, spasticity, tremor)
- ☐ Motor symptoms in legs (weakness, paralysis, coordination, spasticity, tremor)
- ☐ Sensory symptoms (numbness, abnormal sensation, heat intolerance, proprioception - awareness of the position and movement of the body)
- ☐ Visual impairment
- ☐ Speech impairment

- ☐ Difficulty swallowing
- ☐ Respiratory dysfunction
- ☐ Bladder or bowel dysfunction
- ☐ ________ Other (specify):

How often are you assisted by a carer (this could be a professional or partner/family/friend)?

- ○ Never
- ○ Sometimes
- ○ Most or all the time

Do you have difficulty operating a computer or phone?

- ○ Never
- ○ Sometimes
- ○ Often or always

Do you use any of the following assistive technologies regularly?

- ☐ Wheelchair
- ☐ Walking aids (walker, cane, crutches)
- ☐ Bed aids (electric adjustable beds, hoist or lifter)
- ☐ Tools to aid with poor hand dexterity
- ☐ Visual aids and/or vision support cane (white cane)
- ☐ Communication assistance device (text-to-speech)
- ☐ Type assistance device (speech-to-text)
- ☐ Upper limb orthotics
- ☐ Lower limb orthotics
- ☐ ________ Other (specify):

Please list any other activities that you find difficult

Please list any activities that you are concerned you may find difficult in future

The following section discusses technology aimed toward assisting people whose MS symptoms are severe. Please do not continue the survey if you find these topics confronting. You may close the survey at any time.

Scenario Selection

Brain-computer interfaces (BCIs) use sensors to record brain activity and translate that activity into user-intended commands, which can be used to control a device. This project aims to understand what individuals with multiple sclerosis (MS) would like to see in a BCI so that it is most beneficial to them.

Which BCI application would you find most useful in everyday life? If you have no need of these devices now, consider what application you might find most useful in future.

- Wheelchair Control
- Robotic Arm
- Communication

Communication Scenario

Communication Scenario

A friend has asked you if they may come inside your home. Using your brain-computer interface (BCI), you would like to respond with:

“Welcome. Please come in.”

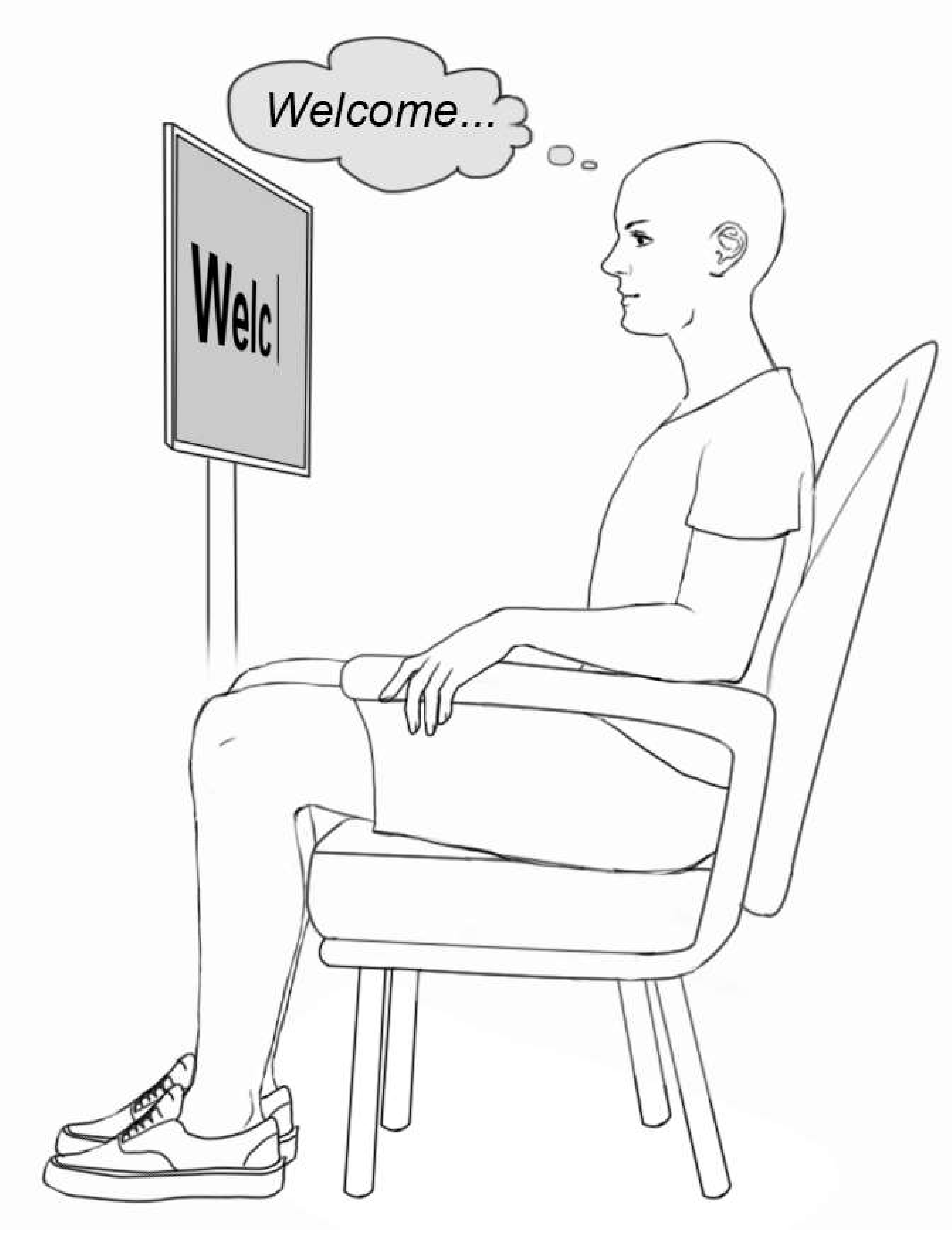

You have the following BCIs available to you, or a carer may help you convey the message with help from cue cards. Please read the information provided carefully.

Please rank these options in order of preference, considering all the information provided.

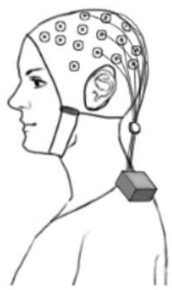

Electrode Cap Brain-Computer Interface (Non-invasive)

You wear a cap over your head. The cap houses several small electrodes. This is painless. Fitting and removing the cap takes approximately 30 minutes each day, and requires assistance. This BCI allows you to communicate the sentence in 1 minute with considerable mental effort.

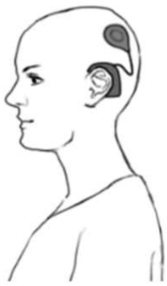

Sub-scalp Electrode Brain-Computer Interface (Invasive)

Several electrodes are implanted beneath your skin on your head. This is a low-risk, painless, day-procedure with no required hospital stay. You wear a behind-the-ear module. There is no further daily setup required. This BCI allows you to communicate the sentence in 1 minute with considerable mental effort.

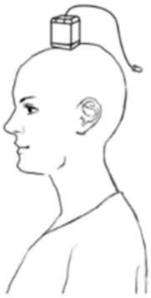

Intracranial Electrode Brain-Computer Interface (Invasive)

Several electrodes are implanted in or directly on your brain. This procedure requires a craniotomy (opening your skull). There is a recovery period in hospital for 1-2 weeks. A small module rests on your head with a cable connecting to the wheelchair. There is no further daily setup required. This BCI allows you to communicate the sentence in 15 seconds with little mental effort.

Carer Assisted

A carer will communicate your intentions for you with the help of cue cards.

Robotic Arm Scenario

Robotic Arm Scenario

You would like to have a drink using your brain-computer interface (BCI) controlled robotic arm. The process involves reaching for the bottle, grasping it, moving it toward you for a drink, returning the bottle to the table and then releasing it.

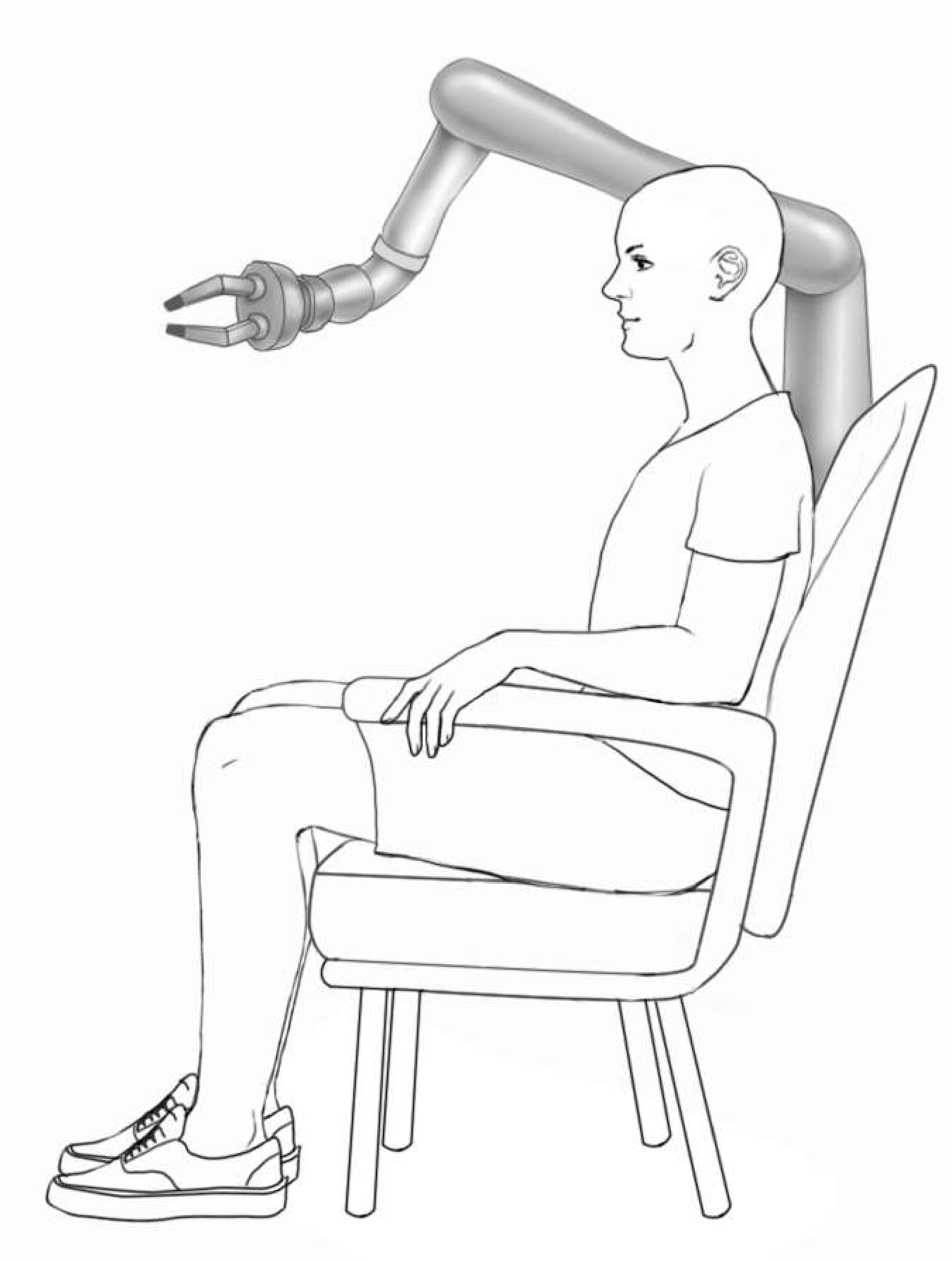

You have the following BCIs available to you, or you may simply ask a carer to perform the task for you. Please read the information provided carefully.

Please rank these options in order of preference, considering all the information provided.

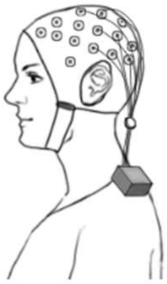

Electrode Cap Brain-Computer Interface (Non-invasive)

You wear a cap over your head. The cap houses several small electrodes. This is painless. Fitting and removing the cap takes approximately 30 minutes each day, and requires assistance. To become proficient with the arm, you will require 10 weeks of training. This BCI allows you to perform the task in 3 minutes with considerable mental effort.

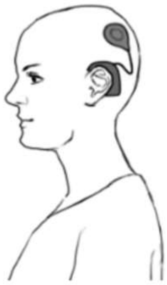

Sub-scalp Electrode Brain-Computer Interface (Invasive)

Several electrodes are implanted beneath your skin on your head. This is a low-risk, painless, day-procedure with no required hospital stay. You wear a behind-the-ear module. There is no further daily setup required. To become proficient with the arm, you require 10 weeks of training. This BCI allows you to perform the task in 3 minutes with conisderable mental effort.

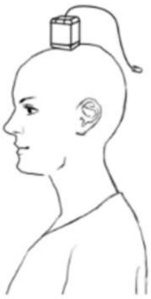

Intracranial Electrode Brain-Computer Interface (Invasive)

Several electrodes are implanted in or directly on your brain. This procedure requires a craniotomy (opening your skull). There is a recovery period in hospital for 1-2 weeks. A small module rests on your head with a cable connecting to the wheelchair. There is no further daily setup required. To become proficient with the arm, you require 2 weeks of training. This BCI allows you to perform the task 1 minute with little mental effort.

Carer Assisted

A carer will lift the bottle for you.

Wheelchair Control Scenario

Wheelchair Control Scenario

You are trying to get from your room to the elevator in your brain-computer interface controlled wheelchair. Your path is outlined below. There are doorways, turns, corridors and obstacles. The distance you must travel is 20m.

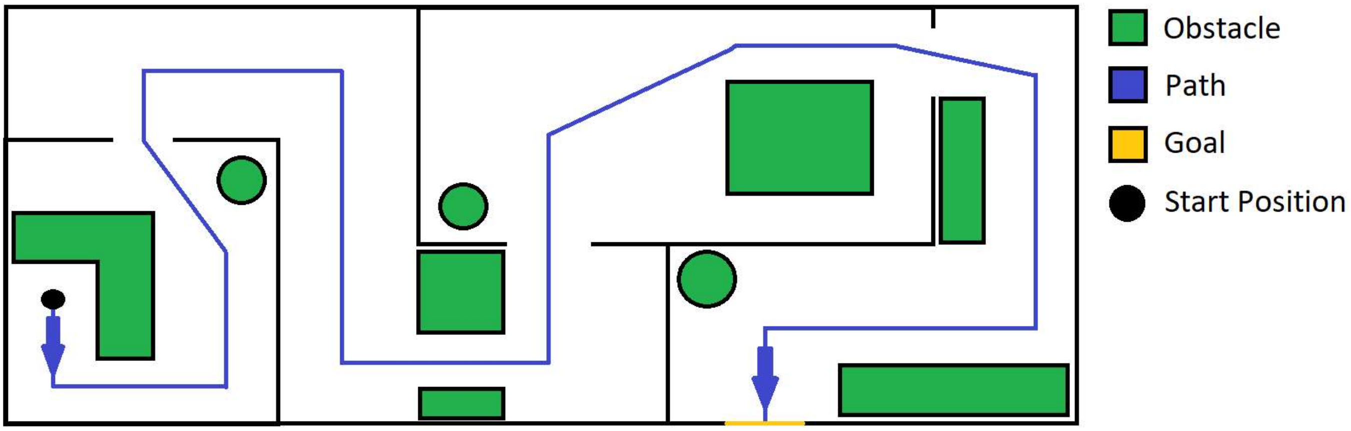
Obstacle
Path
Goal
Start Position

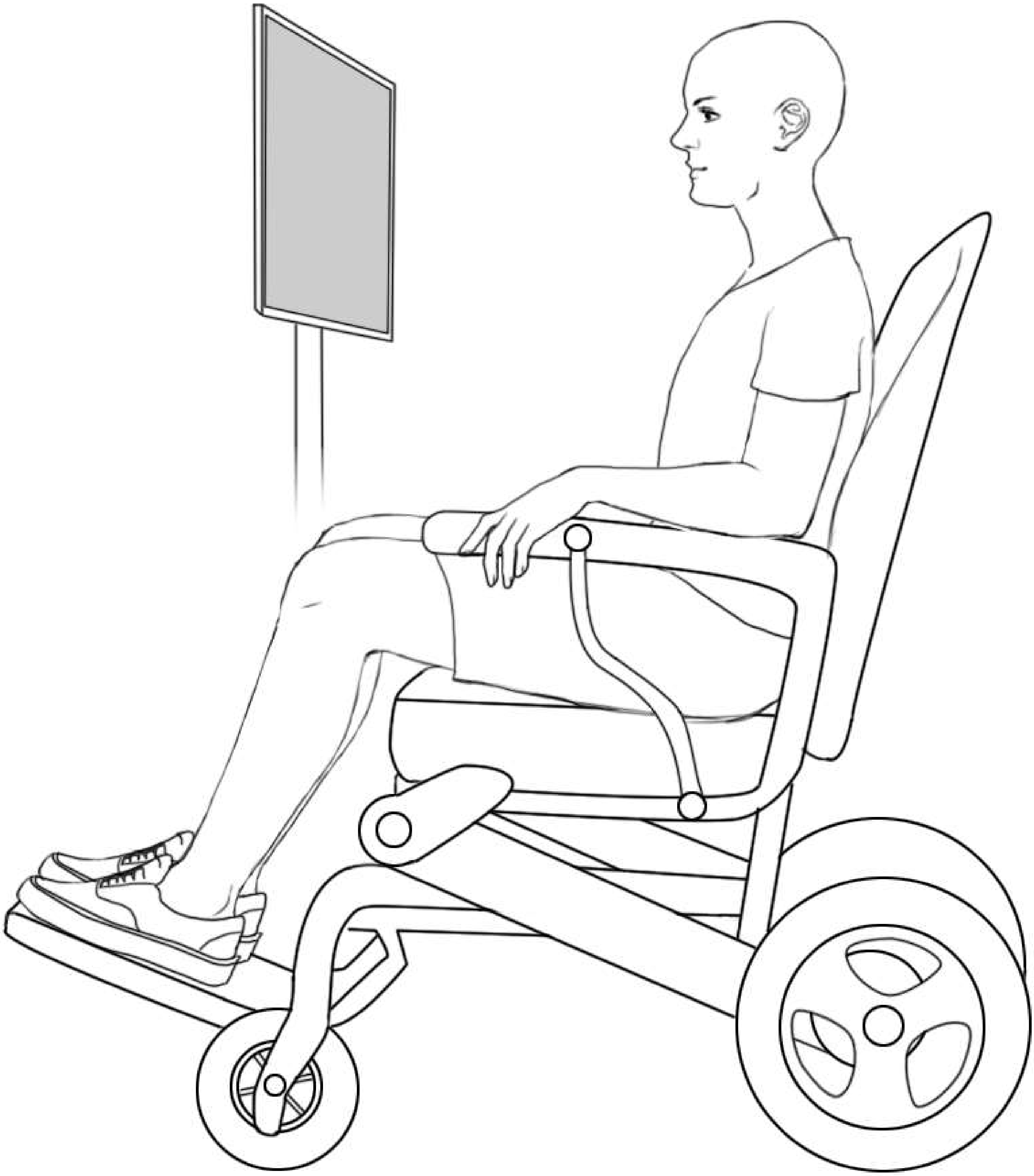

You have the following BCIs available to you, or you may simply ask a carer to perform the task for you. Please read the information provided carefully.

Please rank these options in order of preference, considering all the information provided.

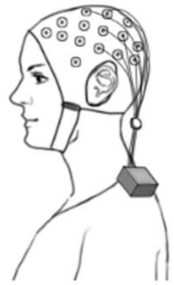

Electrode Cap Brain-Computer Interface (Non-invasive)

You wear a cap over your head. The cap houses several small electrodes. This is painless. Fitting and removing the cap takes approximately 30 minutes each day, and requires assistance. To become proficient with the wheelchair, you will require 4 weeks of training. This BCI allows you to navigate the course in 10 minutes with considerable mental effort.

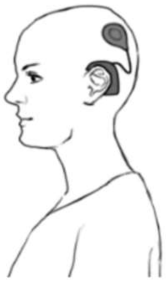

Sub-scalp Electrode Brain-Computer Interface (Invasive)

Several electrodes are implanted beneath your skin on your head. This is a low-risk, painless, day-procedure with no required hospital stay. You wear a behind-the-ear module. There is no further daily setup required. To become proficient with the wheelchair, you require 4 weeks of training. This BCI allows you to navigate the course in 10 minutes with considerable mental effort.

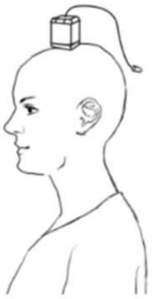

Intracranial Electrode Brain-Computer Interface (Invasive)

Several electrodes are implanted in or directly on your brain. This procedure requires a craniotomy (opening your skull). There is a recovery period in hospital for 1-2 weeks. A small module rests on your head with a cable connecting to the wheelchair. There is no further daily setup required. To become proficient with the wheelchair, you require 2 weeks of training. This BCI allows you to navigate the course in 3 minutes with little mental effort.

Carer Assisted

A carer will push you to the destination.

Alternative Bionic Technologies

Bionic devices may be able to assist with other aspects of MS. Which of the following applications would you find most useful?

- ○ BCI scenario choice
- ○ Bladder control
- ○ Bowel control
- ○ Sexual function
- ○ Vision assistance
- ○ Hearing assistance
- ○ Swallowing assistance
- ○ Sensory assistance
- ○ Temperature regulation

Final Consent

By submitting this survey you consent to your responses being included in the study. If you do not wish your responses to be recorded, please close the survey now.

Powered by Qualtrics
